# Transferring of clubroot-resistant locus *CRd* from Chinese cabbage (*Brassica rapa*) to canola (*Brassica napus*) through interspecific hybridization

**DOI:** 10.1270/jsbbs.21052

**Published:** 2022-06-24

**Authors:** Zongxiang Zhan, Nadil Shah, Ru Jia, Xiaonan Li, Chunyu Zhang, Zhongyun Piao

**Affiliations:** 1 College of Horticulture, Shenyang Agricultural University, Shenyang 110866, Liaoning, China; 2 National Key Laboratory of Crop Genetic Improvement and College of Plant Science and Technology, Huazhong Agricultural University, Wuhan 430070, China

**Keywords:** clubroot resistance, *Brassica napus*, interspecific hybridization, marker-assisted selection

## Abstract

Clubroot, caused by *Plasmodiophora brassicae* is one of the most severe threats to brassica species in China and worldwide. Breeding for clubroot resistant varieties is one of the best ways to overcome this disease. In this study, we introduced clubroot resistance (CR) gene *CRd* from Chinese cabbage (85-74) into elite *Brassica napus* inbred line Zhongshuang 11 through interspecific hybridization and subsequent backcrossing with whole-genome molecular marker-assisted selection (MAS). The resistant test of *CRd* to *P. brassicae* isolates was evaluated in the greenhouse as well as in field conditions. Close linkage markers and the whole-chromosome background marker selection approach improved the recovery rate from 78.3% in BC_1_ to 100% in BC_3_F_1_. The improved clubroot-resistant variety, Zhongshuang11R, was successfully selected in the BC_3_F_2_ generation. The greenhouse and field resistant tests revealed that Zhongshuang11R was resistant to *P. brassicae* pathotypes. The agronomic characteristics of Zhongshuang11R were similar to those of its recurrent parental line, including oil content, composition of fatty acid, plant height, primary effective branches, grain yield per plant and thousand-seed weight. In addition, the oil quality could satisfy the quality requirements for commercial rapeseed oil. Our results will enrich the resistant resources of canola and will certainly accelerate clubroot resistance breeding programs in *B. napus*.

## Introduction

Clubroot disease caused by the obligate biotroph *Plasmodiophora brassicae* Woronin, is one of the most destructive and oldest diseases worldwide of *Brassica* crops and vegetables causing 10–15% yield reduction (Diederichsen *et al.* 2009, Dixon 2009). The disease is widely spread to canola crop (*Brassica napus*) in Europe, Canada and China and production losses increased by 30% (Donald and Porter 2014, Zhan *et al.* 2015).

In China, the disease is widely spreading in canola growing provinces, especially in Sichuan, Hubei, Yunnan and Anhui province (Shah *et al.* 2019). Since it was reported, several pathotypes (P1, P2, P4, P7 and P10) of *P. brassicae* have been identified in the canola field and among these, P4 is the most predominant race found in China, mostly in Hubei and Sichuan provinces (Ren *et al.* 2014, Zheng *et al.* 2018). The genetic diversities among *P. brassicae* isolates are very high (Shah *et al.* 2019) and challenging for canola production.

The causal agent of clubroot is a soil borne pathogen and the resting spores can survive up to 20 years in soil (Donald and Porter 2009, Kageyama and Asano 2009), making it difficult to successfully manage by cultural, chemical and biological practices once the soil is contaminated. Therefore, development of resistant *Brassica* cultivars is one of the most effective, economical and eco-friendly approaches to control the disease, as the other management strategies are non-effective. *Brassica rapa* contains the majority of resistant sources and clubroot resistant Chinese cabbage successfully grown in China, Japan and Korea (Kim *et al.* 2016, Kuginuki *et al.* 1999, Piao *et al.* 2007). Thus, enhancing clubroot resistance (CR) in canola oilseed crops is one of the major canola breeding objectives. In Europe, two resistant cultivars, Mendel and Tosca were developed and released in Germany (Rahman *et al.* 2011). The clubroot resistant in winter canola cv. ‘Mendel’ is originated from resynthesized *B. napus* (AACC, 2n = 38) line that was developed through crossing of the resistant *B. oleracea* line ‘ECD-15’ (CC, 2n = 18) and the resistant *B. rapa* line ‘ECD-04’ (AA, 2n = 20) (Diederichsen and Sacristan 1996). In Canada, researchers introgressed CR into Canadian spring canola from ‘Mendel’ and developed several lines showing high resistance to Canadian *P. brassicae* pathotypes (Rahman *et al.* 2014). In China, several resistant cultivars with ECD04 background was successfully cultivated showing resistance to different pathotypes (Zhan *et al.* 2015).

Breeding for resistant cultivars requires resistant resources and successfully introgression of these resistance genes or loci into new cultivars. Among the A and C genomes of *Brassica* species, some *B. rapa* lines (2n = 20, AA) possess majority CR locus and exhibit higher resistance than *B. oleracea* lines (2n = 18, CC) (Hasan *et al.* 2012, Rahman *et al.* 2011). In *B. rapa*, resistant resources are found most commonly in European fodder turnip (*B. rapa* spp. rapifera) cultivars such as ‘Gelria R’, ‘Siloga’, ‘Debra’ and ‘Milan White’ and in the European Clubroot Differential host ‘ECD-04’. More than 17 resistant loci have been mapped on A1, A2, A3, A5, A6 and A08 (Hejna *et al.* 2019). However, only *CRa* and *Crr1a* are successfully cloned and mapped on chromosome A3 and A8, respectively (Hatakeyama *et al.* 2013, Ueno *et al.* 2012).

Interspecific hybridization is a powerful tool for the improvement of crop species, and it has the potential to enhance genetic diversity and create new plant forms for breeding programs (Chandra *et al.* 2004, Zhan *et al.* 2017). Several important traits, such as blackleg disease resistance and the restorer gene for the Ogura-INRA cytoplasmic male sterility system, have been introgressed into *B. napus* through interspecific hybridization (Pellan-Delourme and Renard 1988, Yu *et al.* 2012). Although, the CR traits are successfully introgressed into *B. napus*, but there are limited resistant sources available (Rahman *et al.* 2011). Thus, CR breeding in canola is still challenging, especially because of genetic diversity in pathotypes of *P. brassicae* field populations.

Therefore, this study was designed to introduce the CR gene *CRd* (Pang *et al.* 2018) from Chinese cabbage into the elite *B. napus* inbred line Zhongshuang 11 by interspecific hybridization and subsequent backcrossing combined with whole-genome molecular marker-assisted selection (MAS). The advanced clubroot-resistant cultivars developed in this study will facilitate the introgression of CR into other Chinese canola cultivars.

## Materials and Methods

### Plant materials and Technical route

**Fig. 1.**
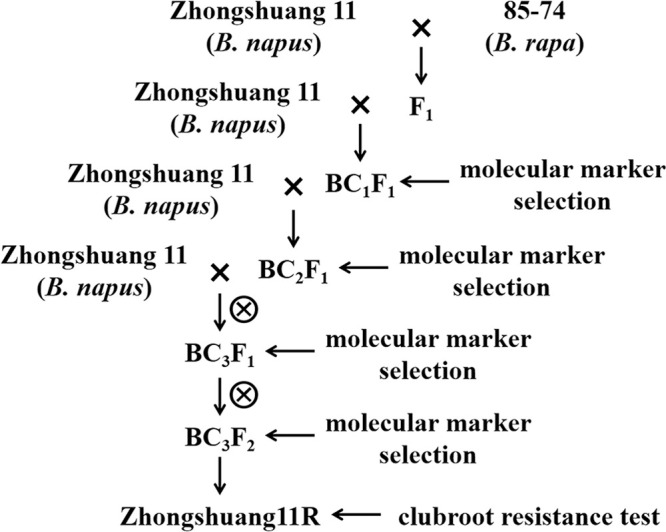
Flow chart of Zhongshuang11R clubroot resistant variety containing *CRd*.

A novel clubroot resistance gene, *CRd*, was fine mapped on chromosome A03 in the Chinese cabbage (*B. rapa*) inbred line 85-74, which was used as the CR donor parent for interspecific gene introgression. An interspecific cross was made between 85-74 (*B. rapa*) and the winter-type canola cultivar Zhongshuang 11 (*B. napus*) to produce an allotriploid F_1_. Subsequently, the allotriploid F_1_ hybrid was backcrossed with Zhongshuang 11 as the female parent several times to produce backcross posterities. The individual with the highest recovery rate was backcrossed with Zhongshuang 11 after selection with the close linkage markers of *CRd* (Fig. 1).

### Marker-assisted selection (MAS)

DNA extraction, PCR amplification and SSR marker analysis was performed as the procedure described previously by Pang (Pang *et al.* 2018). Chi-square analysis was performed in all the backcross and selfing generations to determine segregation of the SSR markers that were linked to the *CRd*. The close linkage markers of *CRd*-assisted selection, YAU78, YAU122 (Supplemental Table 1) were used in each of backcross and selfing populations (Pang *et al.* 2018). A total of 83 SSR markers distribution on the 10 chromosomes of *B. rapa* were employed in the earlier generations, and the marker numbers decreased with increasing backcross generations.

### Clubroot resistance evaluation

The Chinese cabbage donor parent 85-74 and canola cultivar Zhongshuang 11 were evaluated for clubroot disease by inoculation with *P. brassicae* field isolates collected from highly infested fields in Hubei, Anhui, Sichuan and Yunnan provinces of China. These provinces are the main winter-type canola producing regions. Clubroot screening was carried out in greenhouse conditions. Resting spores were extracted from homogenized club roots and diluted to a density of 10^7^ spores per milliliter with sterile distilled water (Xue *et al.* 2008). One-week-old tested plants were inoculated with diluted resting spores by injection into the soil around the plant root zone according to the method described by Chen (Chen *et al.* 2013). The inoculated plants were kept in the greenhouse maintaining day/night temperature at 25/20°C (Shah *et al.* 2019). And the light intensity was 350 μmol·m^–2^·s^–1^. After 6 weeks of inoculation, plants were carefully uprooted and roots are thoroughly washed, and primary and secondary roots were assessed for gall formation. The severity of disease was calculated on a 0 to 3 scale, where 0 = no galls on roots; 1 = few small galls on secondary roots; 2 = small galls on both primary and secondary roots; and 3 = many large galls on both primary roots (Pang *et al.* 2018, Suwabe *et al.* 2006). Disease severity scores of 0 and 1 was considered resistant, and scores of 2 and 3 were considered susceptible plants.

### Field trials and trait evaluation

The developed clubroot resistant line Zhongshuang11R together with its recurrent parents were grown in two consecutive years in 2018–2019 and 2019–2020 in winter-type oilseed rape growing season at Jixi field without *P. brassicae* for agronomic traits evaluation. The field trials were designed at complete randomized design with three replications. The planting methods were selected direct-sowing seed drill and the planting density was about 25 plants/m^2^ after training at seeding stage. The area of each plot was about 10 square meters. Ten randomized individuals in each replicate were used for morphological characteristics evaluation, and the cleaned seeds after air-dried were employed for oil quality and thousand seed weight analysis. Field trials for clubroot resistant tests were carried out at two different places, Xinmin Liaoning province and Jixi Anhui province.

### Oil content and composition analysis

To determine the seed oil content and composition, the gas chromatography (GC) technique was employed (Anand and Downey 1981). About 3 g of dried canola seeds was ground in a mortar. Then, a portion of the ground seeds (0.5 mg) was transferred into a 5 mL glass tube. 1.5 mL of 2.5% methanol sulfate solution and 350 μL of methylbenzene were added, the glass tubes were carefully sealed and kept at 90°C in a hot water bath for approximately 30–45 minutes. After cooling at room temperature, 1 mL of double-distilled H_2_O and 1 mL of *n*-hexane was added into each tube, mixed and centrifuged at 1000 rpm for 5 min (Thermo Fisher Scientific, Germany). The supernatant of fatty acid methylated ester was removed to autosampler vials, and 0.5 μL of sample was injected and analyzed by GC (HP7890A, Agilent) with a nitrogen carrier gas flow rate of 30 mL/min. The initial oven temperature was 180°C for 2 min, followed by 10°C/min to a final temperature of 220°C, which was held for 12 min. Three samples of rapeseed were used as testing materials.

The fatty acid standards including palmitic acid (C16:0), stearic acid (C18:0), oleic acid (C18:1), linoleic acid (C18:2), linolenic acid (C18:3), eicosenoic acid (C20:1) and erucic acid (C22:1) were purchased from Sigma-Aldrich Shanghai, China. The mean value and standard deviation from three biological replicates were calculated. Quantification of each fatty acid composition was carried out by the percentage of peak values by using corresponding standard samples (Zhang *et al.* 2013).

### Statistical analysis

Statistical analysis was performed with SPSS 20 software. The Graph Pad 5 prism software was used to construct the graphs.

## Results

### Clubroot disease evaluation of parental lines

In the greenhouse, we assessed clubroot resistance test of the 85-74 and Zhongshuang 11 by inoculating of 9 different *P. brassicae* isolates collected from four different provinces of China (Table 1). The inbred line 85-74 containing *CRd* resistant gene that showed resistance to all field pathotypes. Only two individuals were recorded small galls on lateral roots when inoculated with Yunnan-KM isolates with the disease index 3.92 (Table 1). However, the recurrent parent Zhongshuang 11 exhibited susceptibility against all field isolates and the disease indices ranging from 76.67 to 100 (Table 1). These results indicating that *CRd* is resistant to most pathotypes of the main canola cultivation regions in China and has great potential application value in the CR breeding of canola.

### Interspecific hybridization and MAS

An interspecific cross-pollination was made between the *B. napus* canola cultivar Zhongshuang 11 (AACC) and the *B. rapa* Chinese cabbage inbred clubroot resistant line 85-74 to form a combination of allotriploid F_1_ hybrid (Zhongshuang 11 × 85-74). The flanking markers (YAU78, YAU122) were used to screen the presence of CR gene and susceptible alleles in the backcross populations. Chi-square tests (χ^2^) revealed that the genotype segregation fit at 1:1 ratio for the two markers in the backcross population (χ^2^ = 0.02–2.13 < χ^2^0.05(1) = 3.84) and 1:2:1 ratio fit in BC_3_F_2_ generation (χ^2^ = 1.20–1.62 < χ^2^0.05(2) = 5.99) (Table 2). These results indicated that the transmission of *CRd* included a chromosomal region in which no segregation distortion was observed.

**Fig. 2.**
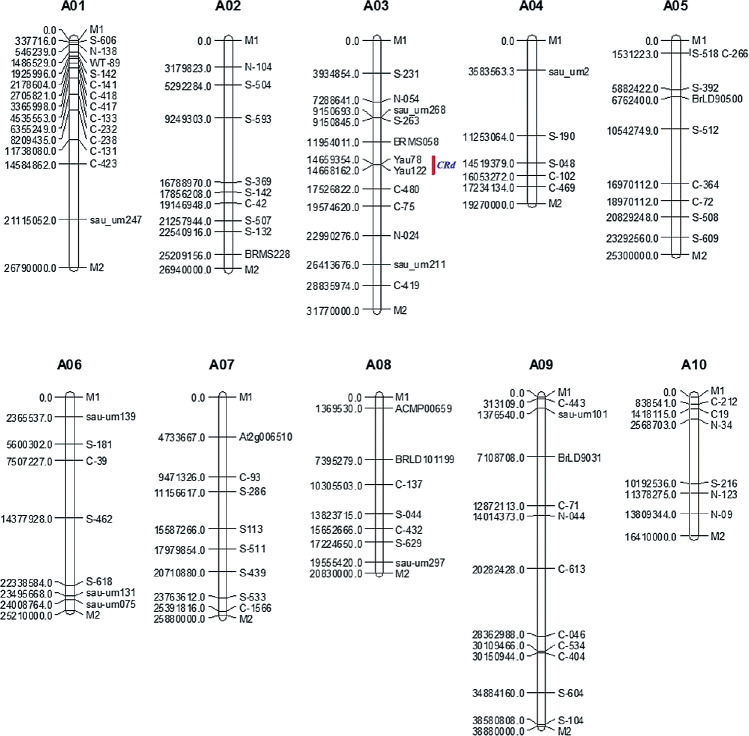
Physical map of the markers used in this study. M1 and M2 represent the starting site and ending site of each chromosome.

To accelerate the breeding progress, a total of 83 polymorphic markers were used for background screening. All markers were distributed on 10 chromosomes of the A genome. The average physical distance between adjacent markers was approximately 3.06 MB (Fig. 2). The chromosomes with the highest and fewest numbers of molecular markers (13 and 5, respectively) were A01 and A04. All individuals with the *CRd* locus confirmed by YAU78 and YAU122 were screened with these background markers. The genetic background recovery rates increased dramatically from BC_1_F_1_ to BC_3_F_2_ generation. In the BC_1_F_1_ generation, 30 individuals with the highest background recovery rates (78.3%) were used for backcrossing with Zhongshuang 11. Due to the limited number of individuals, we only screened the individuals with the highest recovery rates (93.7%) in the BC_2_F_1_ generation (Table 2). Similarly, in the BC_3_F_1_ generation, the highest recovery rates (100%) individual was successfully selected and used to produce the BC_3_F_2_ generation. In this generation, individuals with a recovery rate of 100% and Homozygous resistance sites were successfully screened, after selection with linkage markers YAU78 and YAU122. To produce pure-breeding canola genotypes, homozygous materials with dominant alleles for CR were successfully selected from the descendants with recovery rates of 100% (Table 2).

### Clubroot resistance test of the improved *B. napus*

**Fig. 3.**
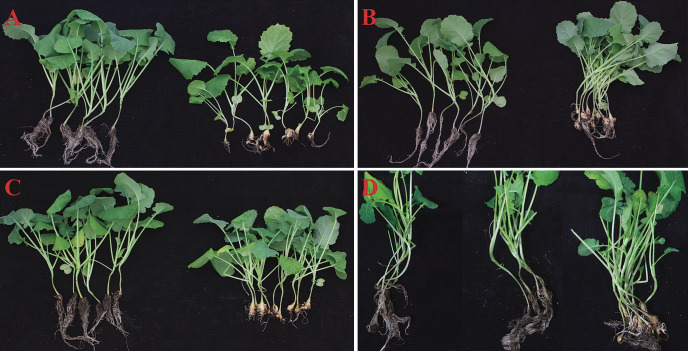
Clubroot resistance test of Zhongshuang11R and Zhongshuang 11. Root symptom evaluation of Zhongshuang11R (left) and control material Zhongshuang 11 individuals (right). A–D show inoculation with the pathogenic races of *P. brassicae* from Yunnan-KM, Anhui-SH Hubei-HY, and Sichuan-MY, respectively. D: The individuals in the middle are Zhongshuang11R with small clubs.

The improved inbred line screened from the BC_3_F_2_ generation was named Zhongshuang11R. To confirm its resistance characteristics, greenhouse inoculation and field disease resistant tests were employed. In the greenhouse test, Zhongshuang11R was inoculated with four different *P. brassicae* field populations, collected from canola main producing regions (Supplemental Table 2). The resistant test revealed that the Zhongshuang11R showed 100% against the pathotypes collected from Anhui, Hubei and Yunnan provinces at 45 days after inoculation (Fig. 3). Whereas a minor infection (2.08%) was observed in the lateral roots to Sichuan-MY isolate (Supplemental Table 2). On the other hand, the Zhongshuang 11 showed high susceptibility (97.26 to 100%) against all pathotypes (Supplemental Table 2).

For the field experiment, both Xinmin, Liaoning province and Jixi, Anhui province were selected for the CR screening, which were heavily infested by *P. brassicae*. The parental line Zhongshuang 11 showed a high rate of disease index and incidence (94.52 and 97.26%), respectively (Supplemental Table 2). However, the disease index and incidence rate in Zhongshuang11R was significantly lower (3.23 and 9.68%, respectively) than that compared with Zhongshuang 11 in Xinmin. The same results were also observed in the two consecutive planting seasons in Jixi, Anhui province (Supplemental Table 3). In two continue planting seasons, the disease index of Zhongshuang11R was lower than 1.05. However, the disease index of recurrent parental line Zhongshuang 11 was more than 62.44. Taken together, these results demonstrated that the improved material Zhongshuang11R showed an excellent resistance to clubroot isolates.

### Morphological characteristics analysis and evaluation of Zhongshuang11R

**Fig. 4.**
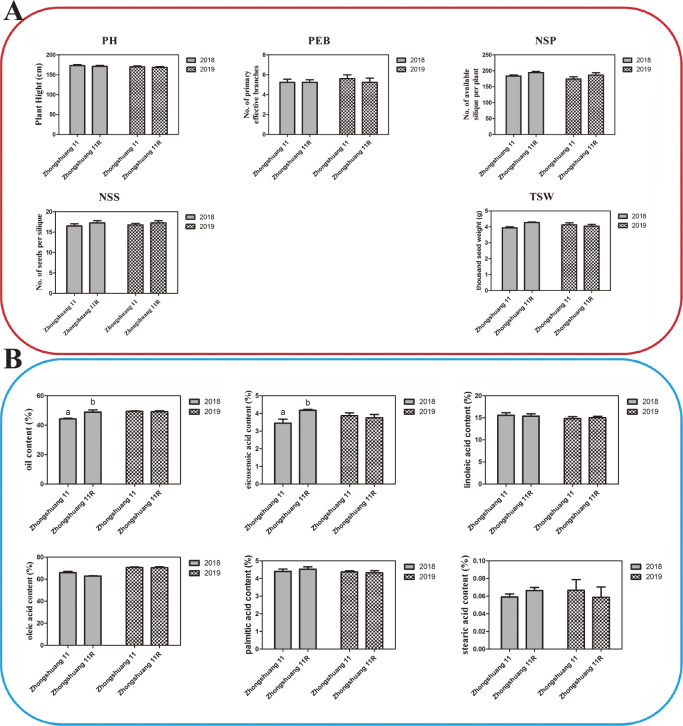
Morphological characteristics evaluation and oil content and composition analysis of seeds analysis between Zhongshuang11R and Zhongshuang 11. A: Morphological characteristics evaluation. Error bars represent + SE calculated from 10 independent individuals in three replicates. B: Oil content and composition analysis of seeds. Error bars represent + SE calculated from three replicates. Statistically significant differences are calculated with Student’s t-test and indicated by letter (P-value <0.05).

Although the recovery rate of Zhongshuang11R was 100% based on molecular marker information, the morphological characteristics and fatty acid composition of Zhongshuang11R was carried out in two consecutive years in 2018–2019 and 2019–2020. The morphological characteristics of Zhongshuang11R were almost the same as that of its parental line during the vegetative and generative stages in two consective growing season (Supplemental Fig. 1). The leaf type of Zhongshuang11R was most typical to Zhongshuang11. Leaf-blades had a gray-green color with serrated edges (Supplemental Fig. 1A). The character of bolting stem and flower were almost the same between the two materials (Supplemental Fig. 1B, 1C). There were no significant differences observed in the plant height (PH), primary effective branches (PEB) and number of siliques per plant (NSP) between these two materials in the two growing seasons (Fig. 4A). The thousand-seed weight (TSW) and number of seeds per silique (NSS) of Zhongshuang11R was slightly higher than that of the parental line in the first growing season (Fig. 4A). However, this marginal deviation was not observed in the second growing season, which was probably related to the environmental variation.

The seed oil quality and the oil content in the seed of *B. napus* are important criteria in breeding programs. Therefore, GC was employed to evaluate fatty acids composition in Zhongshuang 11 and Zhongshuang11R. The average oil content in Zhongshuang11R was higher than that of Zhongshuang 11 in the first growing season, which was 48.46% and 44.30%, respectively (Fig. 4B). This variation was related to unsaturated fatty acid content. Similarly, the average eicosenoic acid content in Zhongshuang11R was 4.18%, which was significantly higher than Zhongshuang 11. However, in the second growing season, no significant differences were observed between Zhongshuang 11 and Zhongshuang11R. The average oil content of Zhongshuang 11 was about 49.92% and was Zhongshuang11R 49.10% and, the average eicosenoic acid content was 3.75% and 3.87%, respectively (Fig. 4B). The clubroot disease intensity probably causes the deviation because *P. brassicae* reduces productivity and also lowers the grain quality (Supplemental Table 3). The other fatty acid contents, like, including linoleic acid, oleic acid, stearic acid and palmitic acid were not significantly difference in both growing seasons (Fig. 4B). The erucic acid content in Zhongshuang11R was zero, as expected, which was the same as its recurrent parental line. Overall these results indicating that the Zhongshuang11R line satisfies the quality requirement for commercial rapeseed oil.

## Discussion

Clubroot, caused by the biotrophic soil-borne pathogen *P. brassicae* that reduces the quality and quantity of important oilseed crop worldwide. In recent years, the disease is rapidly spreading in canola grown in China, causing substantial economic losses in production. The breeding of highly resistant varieties is the most effective way to control this disease (Diederichsen *et al.* 2009). However, resistant resources are extremely limited in *B. napus*. A total of 94 *B. napus* accessions were screened using pathotype 3 in Canada, but only one resistant resource has been identified (Liu *et al.* 2018, Peng *et al.* 2014). Fortunately, The CR materials have been identified in other *Cruciferae*, such as *B. rapa*, *B. oleracea*, and *B. rahanus*. Some CR loci have been successfully introduced into *B. napus* from *B. juncea* or *B. rahanus* by using complicated protocols (Liu *et al.* 2018, Zhan *et al.* 2015). However, these methods cannot satisfy the imperious needs of CR resources in canola breeding, because of the genetic diversity of clubroot pathogen. European fodder turnip (*B. rapa* ssp. *rapifera*) carries clubroot resistant genes against a number of *P. brassicae* isolates (Crute *et al.* 1983, Hasan *et al.* 2012). Therefore, it is significantly important to fully utilize the CR resources of *B. rapa* will greatly promote the progress of CR breeding in canola through interspecific hybridization between *B. rapa* and *B. napus*.

In the current study, *CRd* mapped on the chromosome A03 of the *B. rapa* successfully introgressed into *B. napus* through interspecific hybridization and MAS. The Zhongshuang11R showed great resistance to *P. brassicae* in the greenhouse as well as in the field disease tests. It is possible that the improved line will significantly relieve the pressure caused by the clubroot disease in China.

The identification of new resistant loci and introgression of these sources for CR breeding are important in canola. Pathotype 4 is the most dominant and prevailing *P. brassicae* isolate present in Brassica growing regions (Wang *et al.* 2014). Perhaps, this could be the reason that the line showed resistance to number of isolates collected from different provinces of China. However, in the greenhouse experiments, minor galls were observed upon inoculation with Yunnan-KM, Yunnan-TC and Hubei-NS. A similar kind of results was also observed in the field resistant test with Zhongshuang11R. The uneven distribution, mixture of clubroot pathogen and variance in virulence in the infested field could be the cause of the discrepancy. However, further study is required to confirm.

Interspecific hybridization is a powerful tool for the improvement of crop species, and it has the great potential to broaden the genetic base and create new plant forms for breeding programs. However, sometimes undesirable and desirable traits closely link to each other in the alien segment from the donor that integrates into the genome of the receptor. Usually, it takes a long time to break linkage drag, especially when large genome variation exists between the two materials. For example, breaking the linkage drag associated with the high glucosinolate content in seeds when transferring the gene responsible for Ogr-INGA cytoplasmic male sterility from radish into *B. napus* took more than ten years (Primard-Brisset *et al.* 2005). Another resistance locus, *PbBa8.1*, was linked with FAE1, encoding a seed-specific enzyme of β-ketoacyl-CoA limiting erucic acid biosynthesis was broken in three years (Zhan *et al.* 2020). Luckily, *CRd* was not linked to undesirable agronomic traits.

The average oil content in Zhongshuang11R was slightly higher than that of its recurrent parental line on the first growing season, which was probably caused by the clubroot disease. The disease index of Zhongshuang 11 on 2018–2019 season was significantly higher than that of the second growing season. And, it was reported that clubroot disease could affect canola productivity and grain quality (Pageau *et al.* 2006). The morphological characteristics and oil quality of Zhongshuang11R were more similar to its recurrent parent. However, further work is required to explore whether *CRd* is linked with other detrimental genes in *B. napus*.

## Author Contribution Statement

ZZ analyzed the data, performed the experiments, and drafted the manuscript. RJ performed the experiments. SN helped to analyze the data. CZ helped to draft the manuscript. ZP conceived the study, participated in its coordination, and helped to draft the manuscript. All authors read and approved the final manuscript.

## Supplementary Material

Supplemental Figure

Supplemental Tables

## Figures and Tables

**Table 1.** Clubroot resistant evaluation in parental lines with different pathotypes

Cultivars	85-74				Disease index	Zhongshuang 11				Disease index
SCDS Pathotypes	0	1	2	3		0	1	2	3	
Yunnan-KM	15	2	0	0	3.92	0	1	1	10	91.67
Yunnan-TC	18	0	0	0	0	0	3	1	6	76.67
Sichuan-CD	20	0	0	0	0	0	0	0	10	100
Sichuan-MY	20	0	0	0	0	0	0	0	11	100
Anhui-HS	16	0	0	0	0	0	0	0	12	100
Anhui-XC	16	0	0	0	0	1	0	0	10	90.91
Hubei-BY	20	0	0	0	0	2	0	0	8	80.00
Huibei-NS	16	0	0	0	0	0	0	1	12	97.44
Hunan-HY	20	0	0	0	0	1	0	0	10	90.91

Note: Score of clubroot disease symptoms (SCDS). 0 = no galls on roots; 1 = few small galls on secondary roots; 2 = small galls on both primary and secondary roots; and 3 = many large galls on both primary roots. KM, TC, CD, MY, HS, XN, BY, NS, HY was the local city of different province, Kunmin, Tengchong, Chengdu, Mianyang, Huangshan, Xuancheng, Beiyang, Enshi, Hengyang, respectively.

**Table 2.** Analysis of molecular markers for clubroot resistance gene *CRd* introgression in canola using marker-assisted selection in a cross between the clubroot resistant *Brassica rapa* 85-74 and the susceptible *B. napus* cv. Zhongshuang 11

Population	Name of markers	Total	CR resistance allele		Susceptible allele	χ^2^	The highest recovery rate	Individual numbers
			Homozygous	Heterozygosis				
BC_1_F_1_*^a^*	Yau78	72	–	30	42	2.00	78.3%	72
	Yau122	72	–	30	42	2.00		
BC_2_F_1_*^a^*	Yau78	270	–	147	123	2.13	93.7%	270
	Yau122	260	–	146	124	1.79		
BC_3_F_1_*^a^*	Yau78	230	–	118	112	0.16	100%	230
	Yau122	230	–	118	112	0.16		
BC_3_F_2_*^b^*	Yau78	254	68	124	62	0.56	100%	254
	Yau122	254	67	127	60	0.62		

Note: *a*: expected segregation ratio was 1:1. *b*: Expected segregation ratio was 1:2:1 in BC_3_F_2_.
