# Supplementary material for: Transferring of clubroot-resistant locus CRd from Chinese cabbage (Brassica rapa) to canola (Brassica napus) through interspecific hybridization
Source: Breed Sci. 2022 Jun 24;72(3):189–97. doi: 10.1270/jsbbs.21052 (PMC9653189; doi:10.1270/jsbbs.21052)
Supplement: Supplementary file 1 — Supplemental Figure [file 72_189_s1.pdf]

doi: 10.1270/jsbbs.21052

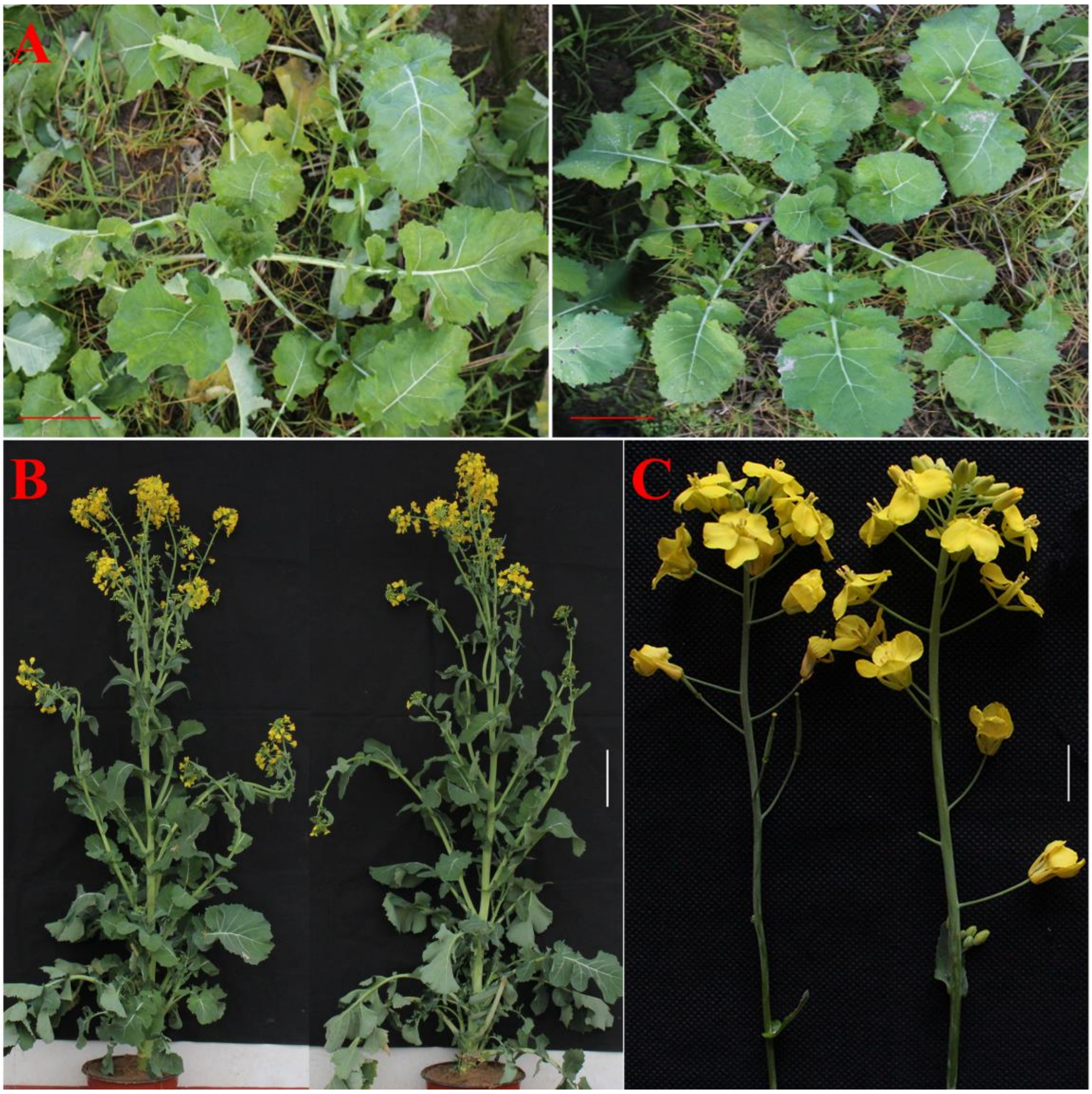

Supplemental Fig. 1. Morphological characteristics of two materials.
A: Seeding stage in the field (bar = 5 cm). B: Full-bloom stage (bar = 10 cm). C. The main trivia of two materials (bar = 2 cm). From left to right was Zhongshuang11R and Zhongshuang 11.
