# Supplementary material for: Transferring of clubroot-resistant locus CRd from Chinese cabbage (Brassica rapa) to canola (Brassica napus) through interspecific hybridization
Source: Breed Sci. 2022 Jun 24;72(3):189–97. doi: 10.1270/jsbbs.21052 (PMC9653189; doi:10.1270/jsbbs.21052)
Supplement: Supplementary file 2 — Supplemental Tables [file 72_189_s2.pdf]

doi: 10.1270/jsbbs.21052

Supplemental Table 1. The linkage primer sequences of *CRd*

Primer name	FORWARD PRIMER1 (5'-3')	REVERSE PRIMER1 (5'-3')	Use
YAU78	AAAGCCGATTCGCCGATCTA	GGCTTTGTTGACGAGAGACC	Selection of resistant locus *CRd*
Yau122	TTGAAACATTCCGGTGCCTT	TCACCCAATCACCATACGAAA	

Supplemental Table 2. Clubroot resistant evaluation of the improved variety Zhongshuang 11R in the greenhouse and field

Cultivars	Zhongshuang 11R				Disease	Incidence	Zhongshuang 11				Disease	Incidence
SCDS / Pathotypes	0	1	2	3	Index	rate(%)	0	1	2	3	Index	rate(%)
Yunnan-KM	45	3	0	0	2.08	6.25	0	1	6	26	91.92	100
Sichuan-MY	36	0	0	0	0	0	0	0	2	36	98.25	100
Anhui-HS	37	0	0	0	0	0	0	0	0	32	100	100
Hunan-HY	39	0	0	0	0	0	0	2	0	35	96.40	100
Xinmin*	56	6	0	0	3.23	9.68	2	0	6	65	94.52	97.26

Note: Score of clubroot disease symptoms (SCDS). 0 = no galls on roots; 1 = few small galls on secondary roots; 2 = small galls on both primary and secondary roots; and 3 = many large galls on both primary roots. KM, MY, HS, HY was the local city of different province, Kunmin, Mianyang, Huangshan, Hengyang, respectively.

* indicates clubroot resistant test in the field.

Supplemental Table 3. Clubroot resistant evaluation of the materials in JiXi, Anhui province.

Cultivars / SCDS / season	Zhongshuang 11R				Disease Index	Incidence rate(%)	Zhongshuang 11				Disease Index	Incidence rate(%)
	0	1	2	3			0	1	2	3		
2018-2019	123	4	0	0	1.05	3.15	15	2	21	89	81.63	81.63
2019-2020	142	2	0	0	0.46	1.39	36	21	19	74	62.44	62.44

Note: Score of clubroot disease symptoms (SCDS). 0 = no galls on roots; 1 = few small galls on secondary roots; 2 = small galls on both primary and secondary roots; and 3 = many large galls on both primary roots.
